# A Comparison of Intranasal Dexmedetomidine, Esketamine or a Dexmedetomidine-Esketamine Combination for Induction of Anaesthesia in Children: A Randomized Controlled Double-Blind Trial

**DOI:** 10.3389/fphar.2021.808930

**Published:** 2022-01-27

**Authors:** Xinlei Lu, Ling Tang, Haiyan Lan, Chunli Li, Han Lin

**Affiliations:** ^1^ Department of Anesthesiology, The Second Affiliated Hospital and Yuying Children’s Hospital of Wenzhou Medical University, Wenzhou, China; ^2^ Department of Anesthesia, Hangzhou Hospital Affiliated with Zhejiang University School of Medicine, Hangzhou First People’s Hospital, Hangzhou, China

**Keywords:** dexmedetomidine, esketamine, intranasal administration, children, preoperative sedation

## Abstract

**Objective:** To compare the efficacy of dexmedetomidine, esketamine or combined intranasal administration on the induction of inhalation anaesthesia in children.

**Methods:** Ninety children aged 1–6 years were randomly allocated into three equal groups to be premedicated with either intranasal dexmedetomidine 2 μg/kg (Group D), esketamine 1 mg/kg (Group S), or dexmedetomidine 1 μg/kg combined with esketamine 0.5 mg/kg (Group DS). The primary endpoint was the Induction Compliance Checklist (ICC) Scale. Secondary outcomes included the sedation success rate; the modified Yale Preoperative Anxiety Scale score; the time of reaching up to two points on the University of Michigan Sedation Scale (UMSS); Parental Separation Anxiety Scale; anaesthesiologist satisfaction with induction based on the visual analogue scale; emergence agitation scale score; and adverse effects.

**Results:** The children in the DS group showed a high degree of cooperation with inhalation anaesthesia induction, and their ICC score was significantly lower than that of the D and S groups (*p* = 0.001), but there was no difference between the D and S groups. The success rate of sedation was higher in Group DS (90%) than in Group D (70%) and Group S (53.3%) (*p* = 0.007). Anaesthesiologist satisfaction with induction was significantly higher in Group DS than in Groups D and S (*p* = 0.001). The incidence of emergence agitation and the Paediatric Anaesthesia Emergence Delirium (PAED) score in the DS group were lower than those in the D and S groups.

**Conclusions:** Preoperative intranasal administration of dexmedetomidine combined with esketamine can significantly improve the cooperation of children with inhalation anaesthesia masks. It is a sedation method that has a high success rate and reduces the incidence and degree of emergence agitation.

## Introduction

Most children undergoing elective surgery are afraid of the hospital environment and the operating waiting area. It is estimated that approximately 60–70% of children show significant anxiety before surgery (Kain et al., 1996). High levels of preoperative anxiety may lead to poor inhalational anaesthesia induction in children and even induction difficulties. The use of violence during inhalation anaesthesia induction is not a suitable option. It may lead to increased demand for postoperative analgesia and emergence agitation or even cause adverse behavioural changes after surgery (Mason, 2017). Therefore, anaesthesiologists should adopt appropriate strategies to reduce the potential psychological trauma to children induced by inhalation anaesthesia. Preoperative sedation is one of the most commonly used methods to prevent and treat preoperative anxiety in children and improve cooperation with inhalation anaesthesia (Kain et al., 2004; Rosenbaum et al., 2009).

Dexmedetomidine is a specific and selective α-2-adrenergic receptor agonist (*α* 2/α 1 = 1,620). It acts on the locus coeruleus and produces sedation similar to physiological sleep. Dexmedetomidine has many characteristics, such as analgesia, sedation (Nelson et al., 2003), anti-sympathetic (Ueki et al., 2014), and anti-inflammation (Venn et al., 2001) effects and no respiratory inhibition. Nasal administration of dexmedetomidine is a common method of preoperative sedation (Baier et al., 2016); however, the drug may lead to bradycardia or hypotension in children (Lei et al., 2020).

Ketamine is a classic anaesthetic. Its main effect is noncompetitive antagonism to N-methyl-d-aspartate (NMDA) receptors. It has sedative and analgesic properties when used at subanesthetic doses. However, it also has some adverse side effects, including nausea, high blood pressure and tachycardia (Tsze et al., 2012; Scheier et al., 2017). Sedation can be given in a variety of ways, including nasal administration. Intranasal infusion of dexmedetomidine combined with ketamine is used for sedation in children (Yang et al., 2019), which has the advantages of stable haemodynamics. Compared to that with dexmedetomidine alone, the onset time is shorter, and it is not easy for patients to wake up. In addition, there is less postoperative agitation and reduced airway secretions compared to those with ketamine alone (Sun et al., 2020).

Esketamine (S (+)-ketamine) is the dextral enantiomer of ketamine. Esketamine is approximately twice as potent as racemic ketamine (Arendt-Nielsen et al., 1996). At the same time, compared with racemic ketamine, esketamine has the properties of a shorter recovery period, less postoperative pain, faster recovery of cognitive function (Himmelseher and Pfenninger, 1998), and lower occurrence of psychiatric side effects (Pfenninger et al., 2002).

Esketamine can counteract the bradycardic and hypotensive effects of dexmedetomidine. Therefore, the combined use of dexmedetomidine and esketamine may be beneficial to patients. However, the preoperative sedation of children with intranasal infusion of dexmedetomidine combined with esketamine or with esketamine alone has not been studied.

This study aimed to compare the effects of dexmedetomidine, esketamine or combined preoperative intranasal infusion on the induction of inhalation anaesthesia in children through a randomized controlled double-blind study to provide a theoretical basis for improving the comfort of anaesthesia in children.

## Materials and Methods

### Research object

This study was a randomized controlled double-blind trial. We obtained approval from the Medical Ethics Committee of The Second Affiliated Hospital and Yuying Children’s Hospital of Wenzhou Medical University. Written informed consent was obtained from all parents/legal guardians. This trial was registered with the Chinese Clinical Trial Registry (registration number ChiCTR2000039445).

The selection criteria were as follows: 1) ASA I-II; 2) scheduled elective lower abdominal or perineal surgery with a surgery time of less than 2 h; and 3) age from 1 to 6 years. 4) The guardian of each child signed the informed consent form.

1.2 The exclusion criteria were as follows: 1) congenital heart disease (tetralogy of Fallot, patent ductus arteriosus, ventricular septal defect, etc.); 2) lung diseases (pulmonary infection, bronchial asthma, etc.); 3) known rhinitis or nasal deformities; 4) allergy to the drugs used in this subject; and 5) known difficult airway.

1.3 The elimination criteria were as follows: 1) laryngospasm during anaesthesia induction; 2) laryngeal spasm after removal of the laryngeal mask; and 3) use of opioids during surgery.

### Research design

The sample size was assessed using NCSS-PASS software version 11.0. According to previous literature (Johansson et al., 2013), the success rate of nasal sedation with 2 μg/kg dexmedetomidine was 69.4%. The noninferiority cut-off value was 15%, *α* = 0.05, *β* = 0.2, and the proportion of patients lost to follow-up was 0.05. The final sample size of this study was 90 patients.

Children who met the inclusion criteria were randomly assigned to three groups, D (n = 30), S (n = 30), and DS (n = 30), using the random number table generated by SPSS 26.0 (SPSS Inc. Chicago, IL, United States). Children were accompanied by their parents into the anaesthesia induction room 40 min before surgery. The child was fasted for more than 8 h, forbidden to have infant formula for more than 6 h, forbidden to have breast milk for more than 4 h, and forbidden to have clear liquids for more than 2 h.

The anaesthesia nurse used an LMA mucosal nebulizer (MAD140, Wolfe-Tory Medical, Inc. Utah, United States) to prepare nasal drops according to the random grouping. The liquid medicine was prepared by the anaesthesia nurse using the LMA^®^ MAD Nasal™ Intranasal Mucosal Atomization Device (Teleflex; Item number: MAD140) according to the randomization. Black opaque tape was used to cover and block the volume of the drug solution and then hand it over to the same anaesthesiologist. Group D received intranasal dexmedetomidine at a dose of 2 μg/kg, group S received 1 mg/kg esketamine, and Group DS received 1 μg/kg dexmedetomidine +0.5 mg/kg esketamine.

Before nasal administration, the anaesthesiologist cleaned the child’s nasal passages with a disposable cotton swab.

Before medication, anaesthesia nurses used the modified Yale Preoperative Anxiety Scale (*m*-YPAS) to evaluate the degree of anxiety in children. The anaesthesiologist uses a mucosal atomization device (MAD) to spray the medicine over a wide range of surface areas of the nasal cavity and then gently massaged the alar nasi to facilitate fluid absorption.

The patient’s parents, the attending anaesthesiologist, the surgeons, and data collection personnel were blinded to the group assignment.

After medication, the anaesthesiologist used the University of Michigan Sedation Scale (UMSS) to observe and record the time when the child reached a UMSS score of two points. At the same time, the respiratory conditions of the children, such as respiratory rhythm, mucocutaneous colour, and lip colour, were closely observed, and adverse reactions of the children after medication were recorded. If there is an adverse reaction after the administration, the adverse reaction shall be recorded in detail and the case shall be excluded.

When the child’s UMSS score reached two points, another anaesthesiologist was taken to the operating room, and the Parental Separation Anxiety Scale (PSAS) was recorded. If the UMSS score of the child did not reach 2 points for more than 40 min, it was considered a failure of sedation, and the satisfactory sedation time was recorded as 40 min.

Upon arrival in the operation room, all patients were monitored with peripheral pulse oximetry. To avoid the influence of monitoring stimulation on evaluation by the anaesthesia Induction Compliance Checklist (ICC) in children, electrocardiography and noninvasive blood pressure monitoring were temporarily withheld.

General anaesthesia was induced with 8% sevoflurane in 100% oxygen at 8 L/min. The anaesthesiologist used a suitable mask (reaching down to the chin and up to the bridge of the nose), gently covered the mouth and nose of the child, and used a pressure measuring valve to control the pressure of the mask to approximately 30 cm H_2_O.

After the child lost consciousness, electrocardiography and noninvasive blood pressure monitoring were performed. At the same time, the sevoflurane concentration decreased from 8 to 4%, the oxygen flow decreased from 8 L/min to 4 L/min, and the concentration remained unchanged. After achieving an adequate depth of anaesthesia, an intravenous line was placed by the operating room nurse, and then the corresponding size laryngeal mask was placed by the anaesthesiologist. The anaesthesiologist evaluated and recorded the child’s ICC score and anaesthesiologist induction satisfaction based on the visual analogue scale (VAS).

After the laryngeal mask was inserted, the corresponding regional anaesthesia was compounded according to our department’s standard. If the child underwent inguinal surgery, an iliohypogastric nerve block with 0.15% ropivacaine +0.8% lidocaine at a dosage of 0.5 ml/kg was used. If the child underwent perineal surgery, caudal anaesthesia with 0.15% ropivacaine +0.8% lidocaine at a dosage of 0.75 ml/kgwas used.

Anaesthesia was maintained using sevoflurane in a 50% oxygen/air mixture at 2 L/min. During the operation, the child maintained spontaneous breathing. The sevoflurane inhalation concentration was adjusted according to the end-tidal carbon dioxide concentration (PetCO2). PetCO2 was maintained at 35–50 mmHg, and the end-tidal concentration of sevoflurane was maintained above 0.7.

At the end of surgery, the laryngeal mask airway was immediately removed, and an inlet pharyngeal airway was placed. Then, the child was sent to the Post Anaesthesia Care Unit (PACU) for observation. The emergence time (stopping sevoflurane until the first eye opening) was observed and recorded by PACU nurses, and the Paediatric Anaesthesia Emergence Delirium (PAED) score was recorded.

### Statistical analyses

All of the individual participant data were analysed using SPSS version 26.0 (IBM SPSS Inc. Armonk, United States). Descriptive statistics were obtained on all the study variables. Data are reported as the means (standard deviations), medians (interquartile ranges, IQRs), or frequencies (percentages) where appropriate. The Kolmogorov–Smirnov test was applied to determine whether continuous variables were normally distributed. Single factor variance analysis was adopted for the comparison of normally distributed quantitative data. Nonnormally distributed data were analysed using the Kruskal-Wallis test. Categorical variables were compared using the χ2 test or Fisher’s exact test, where appropriate. The *p* value was set at 0.05 for statistical significance.

## Results

A total of 105 patients were assessed for eligibility. Finally, 90 patients were enrolled in this randomized study and were randomized into three groups of 30 children each. In Group D, 21 cases were successfully sedated, and 9 cases failed to be sedated. The success rate of sedation was 70%. In Group S, sedation was successful in 16 cases, and sedation failed in 14 cases, of which 1 case had failed sedation and was excluded by the addition of opioids during the operation. The sedation success rate was 55.2%. In Group DS, 27 cases were successfully sedated, and 3 cases showed failed sedation. Among them, one child had failed sedation, and the operation time exceeded 2 h. The sedation success rate was 93.1%. After excluding two children, 30 cases in Group D, 29 cases in Group S, and 29 cases in Group DS were included in the data analysis. The success rate of sedation in Group DS was significantly higher than that in Group D and Group S(χ2 = 10.070,*p* = 0.006<0.05). As shown in Table 1.

**TABLE 1:** Comparison of the intranasal sedation success rate between the groups.

	Success rates
Group D (n = 30)	21 (70%)
Group S (n = 29)	16 (55.2%)
Group DS (n = 29)	27 (93.1%)#
χ2	10.689
*p* Value	0.005

Data are presented as the number (percentage). group D, dexmedetomidine group; group S, esketamine group; Group DS, dexmedetomidine-esketamine group.

*p < 0.05, the dexmedetomidine group versus the esketamine group, **p < 0.05, the dexmedetomidine group versus the dexmedetomidine-esketamine group, #*p* < 0.05, the esketamine group versus the dexmedetomidine-esketamine group.

There were no significant differences between the groups in terms of patient sex, age, weight, preoperative anxiety score (*m*-YPAS), maintenance time of anaesthesia, or duration of surgery, as shown in Tables 2, 3.

**TABLE 2:** Demographic data and preoperative anxiety score.

	Group D (n = 30)	Group S (n = 29)	Group DS (n = 29)	*p* value
Male/female (%)	80.0/20.0	96.6/3.4	93.1/6.9	0.085
Age (months)	41.80 (±18.95)	33.86 (±17.00)	40.17 (±19.14)	0.224
Weight (kg)	15.33 (±3.98)	15.14 (±4.55)	16.83 (±4.47)	0.309
*m*-YPAS	52.50 (45.35–67.00)	58.20 (48.30–73.20)	50.00 (46.60–69.10)	0.456

Data are presented as numbers (percentages), medians (IQRs) or means ± standard deviations; F, female; D, dexmedetomidine; DS, dexmedetomidine-esketamine; M, male; *m*-YPAS, the modified yale preoperative anxiety scale; S, esketamine.

**TABLE 3:** Duration of anaesthesia and surgery.

	Duration of anaesthesia (min)	Duration of surgery (min)
Group D (n = 30)	35.50 (26.25–48.50)	25.50 (14.75–32.00)
Group S (n = 29)	37.00 (27.00–52.00)	25.00 (16.50–42.50)
Group DS (n = 29)	34.00 (30.00–56.00)	23.00 (16.00–38.50)
*p Value*	0.847	0.604

Data are presented as the median (IQR); D, dexmedetomidine; DS, dexmedetomidine-esketamine; S, esketamine.

The time for the child to reach satisfactory sedation was recorded (UMSS score of two points). If the UMSS score of the child did not reach 2 points for more than 40 min, the satisfactory sedation time was recorded as 40 min. The time to satisfactory sedation in Group DS was significantly shorter than that in Group S (*p* = 0.001 < 0.05), but there was no significant difference between Group DS and Group D (Table 4).

**TABLE 4:** Time to satisfactory sedation.

	Time to satisfactory sedation
Group D (n = 30)	23.50 (17.75–40.00)
Group S (n = 29)	28.00 (21.00–40.00)
Group DS (n = 29)	20.00 (15.00–24.00)#
*p Value*	0.001

Data are presented as the median (IQR). D, dexmedetomidine; DS, dexmedetomidine-esketamine; S, esketamine.

*p < 0.05, the dexmedetomidine group versus the esketamine group, **p < 0.05, the dexmedetomidine group versus the dexmedetomidine-esketamine group, #*p* < 0.05, the esketamine group versus the dexmedetomidine-esketamine group.

There was no significant difference in the PSAS among the three groups. The ICC score of Group DS was significantly lower than that of Groups D and S (*p* = 0.001 < 0.05), while there was no difference between Groups D and S. There was a difference between the groups in the anaesthesiologist satisfaction score (*p* = 0.001 < 0.05); Group DS had a significantly higher score than Groups D and S, but there was no difference between Groups D and S, as shown in Table 5.

**TABLE 5:** Comparison of anaesthesia induction.

	PSAS scores	ICC scores	Anaesthesiologist satisfaction score
Group D (n = 30)	1.0 (1.0–2.0)	4.0 (2.75–6.0)	7.0 (6.0–8.0)
Group S (n = 29)	1.0 (1.0–2.5)	4.0 (2.0–6.0)	7.0 (6.0–9.0)
Group DS (n = 29)	1.0 (1.0–1.0)	2.0 (0.0–4.0)*^#^	9.0 (7.5–10.0)*^#^
*p Value*	0.155	0.001	0.001

Data are presented as the median (IQR); D, dexmedetomidine; DS, dexmedetomidine-esketamine; ICC, induction compliance checklist; PSAS, parental separation anxiety scale; S, esketamine.

*p < 0.05, the dexmedetomidine group versus the esketamine group, **p < 0.05, the dexmedetomidine group versus the dexmedetomidine-esketamine group, #*p* < 0.05, the esketamine group versus the dexmedetomidine-esketamine group.

In terms of emergence time, the median emergence time from anaesthesia in Group S was 26.0 min, that in Group D was 35.0 min, and that in Group DS was 33.0 min. There was no significant difference between the three groups (*p* = 0.163 > 0.05). In terms of the quality of emergence, there was a difference in the incidence of emergence delirium between the groups. The incidence of emergence delirium in Group DS was significantly lower than that in Groups D and S (*p* = 0.001 < 0.05). There was a difference in the PAED score between groups (*p* = 0.000 < 0.05). The PAED score of Group DS was lower than that of Group D and Group S, as shown in Table 6.

**TABLE 6:** Emergence time and emergence quality.

	Emergence time (min)	Occurrence of ED	PAED scores
Group D (n = 30)	35.00 (29.75–45.55)	40.0%	8.00 (4.75–12.00)
Group S (n = 29)	26.00 (19.50–41.50)	58.6%	12.00 (6.50–14.50)
Group DS (n = 29)	33.00 (24.00–46.50)	10.3%*^#^	5.00 (3.00–7.50)*^#^
*p Value*	0.163	0.001	0.000

Data are presented as numbers (percentages), medians (IQRs) or means ± standard deviations; D, dexmedetomidine; DS, dexmedetomidine-esketamine; ED, emergence delirium; PAED, paediatric anaesthesia emergence delirium; S, esketamine.

*p < 0.05, the dexmedetomidine group versus the esketamine group, **p < 0.05, the dexmedetomidine group versus the dexmedetomidine-esketamine group, #*p* < 0.05, the esketamine group versus the dexmedetomidine-esketamine group.

Adverse events occurred in six children in Group S. Among them, three children had nausea, one child had increased secretions, one child had separation anxiety, and one child had abnormal irritability. The incidence of adverse events in Group S was significantly higher than that in Groups D and DS (*p* = 0.01 < 0.05), as shown in Table 7.

**TABLE 7:** Adverse events.

	Nausea	Increased secretions	Separation Phenomenon	Abnormal irritability	Total
Group D (n = 30)	0	0	0	0	0
Group S (n = 29)	3	1	1	1	6*
Group DS (n = 29)	0	0	0	0	0^#^
*χ* ^*2*^	—	—	—	—	13.100
*p Value*	—	—	—	—	0.01

Data are presented as numbers. D, dexmedetomidine; DS, dexmedetomidine-esketamine; S, esketamine.

*p < 0.05, the dexmedetomidine group versus the esketamine group, **p < 0.05, the dexmedetomidine group versus the dexmedetomidine-esketamine group, #*p* < 0.05, the esketamine group versus the dexmedetomidine-esketamine group.

## Discussion

The results of this trial showed that children sedated with dexmedetomidine combined with esketamine before surgery had higher cooperation in anesthesia induction, higher success rate of sedation, higher satisfaction of anesthesiologists and lower incidence of agitation after anesthesia. Preoperative sedation with esketamine alone has a low success rate and a high incidence of side effects.

The results of this trial showed that children sedated with dexmedetomidine combined with esketamine before surgery showed greater cooperation with anaesthesia induction, a higher success rate of sedation, higher satisfaction of anaesthesiologists and a lower incidence of agitation after anaesthesia. Preoperative sedation with esketamine alone has a low success rate and a high incidence of side effects.

In our study, it was found that the Group DS [2.0 (0.0–4.0)] inhalation anaesthesia induction coordination score (ICC) was significantly lower than that of Group D [4.0 (2.75–6.0)] and Group S [4.0 (2.0–6.0)], *p* = 0.001 < 0.05. However, there was no significant difference between Group D and Group S. At the same time, compared with Group D and Group S, the satisfaction of anaesthesiologists in Group DS was higher.During the induction of inhalation anaesthesia, the anaesthesiologist puts the anaesthesia mask on the face of the child. Although we used a pressure valve to strictly control the pressure of the mask at approximately 30 cmH2O, the anaesthesia mask still caused compression or even pain in the child’s cheek. This in turn caused the child to wake up from sedation, especially as dexmedetomidine alone produces mild sedation similar to physiological sleep (Baier et al., 2016). Therefore, when receiving anaesthesia mask inhalation induction, children will awaken from a sedative state and fear the induction of anaesthesia, resulting in a decrease in the degree of mask coordination. Esketamine is a noncompetitive antagonist of NMDA receptors that regulates nociceptive sensations. Previous studies have shown that esketamine has a good analgesic effect. Johansson Joakim et al. applied esketamine nasal administration to prehospital analgesia and found that the VAS score decreased from a median of 10 points (8–10) after nasal administration for 5 min to a median of 3 (2–4) points (Johansson et al., 2013). In our study, both dexmedetomidine combined with esketamine intranasal drops or esketamine alone yielded a certain degree of analgesia under the condition of satisfactory sedation, which may help to improve the cooperation of inhalation anaesthesia induction in children. As a result, Group DS using esketamine showed better inhalation anaesthesia induction coordination, lower ICC scores and higher anaesthesiologist satisfaction.

We recorded the time at which the child reached satisfactory sedation (UMSS score of two points). If the UMSS score of the child did not reach 2 points for more than 40 min, the satisfactory sedation time was recorded as 40 min. The time to satisfactory sedation in Group DS was significantly shorter than that in Group S (*p* = 0.001 < 0.05), but there was no significant difference between Group DS and Group D.Previous studies have shown that the plasma concentration of dexmedetomidine reaches the lowest effective sedative concentration after the nasal administration of 2 μg/kg dexmedetomidine for 10 min (Miller et al., 2018). However, the plasma concentration of children after the nasal administration of esketamine reached a peak within 18 ± 13 min (mean ± standard deviation) (Weber et al., 2004). Dexmedetomidine is a specific and selective α-2-adrenergic receptor agonist with a good sedative effect. In addition to being a noncompetitive antagonist of NMDA receptors, esketamine can also stimulate GABA receptors and mediate their anaesthetic effects. The combined application of the two drugs for preoperative sedation in children showed excellent sedative effects.A recent randomized controlled trial by Mang Sun et al. in children with congenital heart disease requiring transthoracic echocardiography showed that intranasal dexmedetomidine combined with ketamine (DEX 2 μg/kg + KET 1 mg/kg) had a faster onset time but longer recovery time than intranasal dexmedetomidine alone (2 μg/kg) (Sun et al., 2020). In this study, the dose of esketamine used in Group S (ESKET 1 mg/kg) was small, and its anaesthetic effect was approximately equal to that of 2 mg/kg ketamine. At the same time, a previous study found that the nasal administration of 3 and 6 mg/kg ketamine cannot achieve sufficient sedation (Tsze et al., 2012).Finally, the success rate of sedation in Group S was low. However, if the UMSS score of the child did not reach 2 points for more than 40 min, we recorded a satisfactory sedation time of 40 min. This may be the reason that the time to satisfactory sedation in the DS group was significantly shorter than that in the S group (*p* = 0.001 < 0.05).Bin Qian et al. compared intranasal dexmedetomidine (2 μg/kg) and dexmedetomidine combined with ketamine (DEX 2 μg/kg + KET 2 mg/kg) for tonsillectomy in preschool children and found that the median onset time of sedation (median) (15 vs 24 min) was significantly faster in the combination group than in the dexmedetomidine-alone group (Qian et al., 2020). In our study, the time to satisfactory sedation in Group D (median) was 23.5 min, which was similar. The formula used in Group DS was DEX 1 μg/kg + ESKET 0.5 mg/kg, and the time to satisfactory sedation (median) was 20 min longer than that observed by Bin Qian et al. This may have been caused by the use of only 1 μg/kg dexmedetomidine in Group DS, which is smaller than the dose of dexmedetomidine used by Bin Qian et al. At the same time, this is also the reason why the time to satisfactory sedation in Group DS was shorter than that in Group D, though there was no significant difference. In our study, the time to satisfactory sedation for children in Group D was shorter than that in Group S, but there was no significant difference. This may have been caused by our small sample size; therefore, we need to further expand the sample size.

In this study, the success rate of sedation in Group S was only 55.2% lower than that in Group D (70%) and Group DS (93.1%). This may have been caused by the low bioavailability of the drug ketamine when administered intranasally (Andolfatto et al., 2013). The concentration of the esketamine preparation we used was 25 mg/ml, and thus, the volume of esketamine used per unit body weight was twice that of dexmedetomidine. A larger drug volume will reduce air exchange in the nasal cavity, causing discomfort or even suffocation in children. This will increase the difficulty of nasal administration or even the failure of the medication and cause swallowing with larger doses of drugs; however, the bioavailability of swallowed esketamine is very low (Grant et al., 1981). Similar to the findings in previous studies, multiple children indicated that the drug had a bitter aftertaste, which may also cause additional irritation, in turn resulting in failure of sedation (Weber et al., 2003; Fantacci et al., 2018). The success rate of sedation in the DS group was 93.1%. In the study of Yang Fei et al., 2 μg/kg dexmedetomidine combined with 1 mg/kg ketamine was used as a nasal sedation regimen, and the success rate was approximately 93% (Yang et al., 2019). The use of 1 μg/kg dexmedetomidine combined with 0.5 mg/kg esketamine in our DS group reduced the dosage of dexmedetomidine, which helped to reduce the incidence of delayed postoperative recovery and accelerated the transport rate in the day ward. This treatment was also useful when extended to outpatient imaging examinations and improved the economic efficiency of the hospital.

Esketamine is considered to be a racemic ketamine that can produce anaesthetic effects, while R (-)-ketamine is considered to produce adverse effects. Esketamine was developed to reduce the psychomimetic side effects of racemic ketamine, but a review showed that the improvement was not obvious (Engelhardt, 1997). Another side effect of ketamine and esketamine is excessive secretion, although the incidence of increased secretion of esketamine is lower than that of ketamine (Adams and Werner, 1997). In our study, a total of six patients (all in group S) experienced adverse effects. The incidence was 20.69%, which was similar to that in previous studies (Marhofer et al., 2001). Among the patients, three children developed nausea, one child had increased secretion, one child developed abnormal restlessness, and one child developed irritability.

In terms of the quality of emergence, we found differences between the incidence of postoperative agitation and PAED scores among the three groups. The incidence of postoperative agitation in Group DS was significantly lower than that in Groups D and S (*p* = 0.001 < 0.05), and the PAED score of Group DS was lower than that of Groups D and S (*p* = 0.000 < 0.05). The preoperative use of esketamine alone for nasal sedation yields a higher incidence of postoperative agitation and higher PAED scores. This may be due to the psychomimetic side effects of ketamine and esketamine. In addition, esketamine can cause sympathetic stimulation, resulting in an increased plasma concentration of norepinephrine (Kienbaum et al., 2001). Although ketamine isomers cause less cognitive impairment than racemic ketamine at equivalent analgesic doses, esketamine can still cause a certain degree of cognitive impairment [38]. This may be the reason why the incidence of postoperative agitation and PAED scores in Group DS were significantly lower than those in Group S. Dexmedetomidine can reduce sympathetic tension and inhibit catecholamine release, and previous studies have shown that it has a good preventive effect on postoperative agitation (Pfenninger et al., 2002). Dexmedetomidine combined with esketamine has a higher preoperative sedation success rate, and esketamine has a certain analgesic ability. Our study showed that Group DS had lower ICC scores, higher mask cooperation, and less fear induction during the induction of anaesthesia. This may be the reason why the incidence of postoperative agitation and PAED scores in Group DS were significantly lower than those in Group D.

## Limitations

Our study has several limitations. First, we do not have a family-centred care plan. Thus, although the preoperative anxiety state of parents may affect the preoperative anxiety level of children, we have not evaluated this aspect, which may affect the sedative effect of preoperative medication. Second, we did not set up different doses of dexmedetomidine in the esketamine test group. Consequently, we cannot evaluate the dose-response relationship of dexmedetomidine combined with esketamine as a preoperative medication. Thirdly, the induction time may exceed 40 min, and some children need longer onset time. Therefore, it should be considered that the guardian will bring the child into the anesthesia induction room 1 hour in advance. Finally, the main result in our study is the success rate of sedation, so we use the success rate as the basis for sample size calculation. But other variables are still bias factors, which should be fully considered in future studies.

Therefore, it is necessary to conduct further research to resolve these limitations.

## Data Availability

The raw data supporting the conclusions of this article will be made available by the authors, without undue reservation.
